# *cj0371*: A Novel Virulence-Associated Gene of *Campylobacter jejuni*

**DOI:** 10.3389/fmicb.2016.01094

**Published:** 2016-07-14

**Authors:** Xueqing Du, Nan Wang, Fangzhe Ren, Hong Tang, Xinan Jiao, Jinlin Huang

**Affiliations:** Jiangsu Key Lab of Zoonosis, Jiangsu Co-Innovation Center for Prevention and Control of Important Animal Infectious Diseases and Zoonoses, Yangzhou UniversityYangzhou, China

**Keywords:** virulence-associated gene, adhesion and invasion, chemotaxis, motility, growth kinetics

## Abstract

*Campylobacter jejuni* is the major cause of human bacterial diarrhea worldwide. Its pathogenic mechanism remains poorly understood. *cj0371* is a novel gene that was uncovered using immunoscreening. There have been no previous reports regarding its function. In this study, we constructed an insertion mutant and complement of this gene in *C. jejuni* and examined changes in virulence. We observed that the *cj0371* mutant showed significantly increased invasion and colonization ability. We also investigated the role of *cj0371* in motility, chemotaxis, and growth kinetics to further study its function. We found that the *cj0371* mutant displays hypermotility, enhanced chemotaxis, and enhanced growth kinetics. In addition, we localized the Cj0371 protein at the poles of *C. jejuni* by fluorescence microscopy. We present data that collectively significantly proves our hypothesis that *cj0371* is a new virulence-associated gene and through the influence of chemotaxis plays a negative role in *C. jejuni* pathogenicity.

## Introduction

The food-borne pathogen *Campylobacter jejuni* is responsible for campylobacteriosis, the most frequently reported food-borne illness in the European Union with ~200,000 human cases reported every year (Aliouane et al., 2016). Its typical clinical signs and symptoms are fever, inflammation, severe abdominal cramping, watery diarrhea and bloody stools, post-infectious sequelae including Guillain Barre Syndrome and other neurological disorders, with a limited morbidity but high mortality (Young et al., 2007; Dash et al., 2015). Poultry is a major source of *C. jejuni*, and only a small proportion of campylobacteriosis cases can be attributed to other animals or environmental sources (Chen and Jiang, 2014). However, the factors and mechanisms that contribute to successful colonization in poultry and virulence in humans remains poorly understood (Chandrashekhar et al., 2015). Despite the availability of both genomic information of different *C. jejuni* strains and genetic tools, the complete understanding of the virulence of *C. jejuni* is still an ongoing effort. In contrast to other diarrhea-causing bacteria, *C. jejuni* does not express a large number of classical virulence factors in human campylobacteriosis (Havelaar et al., 2009). Even so, many atypical virulence factors have been discovered.

With the development of whole genome sequencing technology and bioinformatics, numerous genome sequences of many *C. jejuni* are now available and comparisons identified several new open reading frames that may represent species-specific proteins (Aliouane et al., 2016). Using *in vivo*-induced antigen technology (IVIAT), an immunoscreening method, our group has identified virulence-associated genes expressed during human and chicken infection or invasion by *C. jejuni* (Hu et al., 2014a,b). We successfully identified 48 unique genes expressed *in vivo*, including one novel gene open reading frame. Through sequence blast in NCBI, we confirmed that this novel gene is *cj0371* (Gene ID: 904694, updated on 3-Feb-2016). We speculated it is a novel virulence-associated gene. Because there have not been any reports regarding this gene until now, we were strongly interested in its function.

!!!!! Bioformatics analysis indicated that *cj0371* is highly conserved in *C. jejuni*. This study was carried out to evaluate the ability of a *cj0371* mutant to invade Caco-2 cells, the level of colonization in infant rabbits, and a functional exploration of the *cj0371* gene. A motility plate assay, TEM imaging and subcellular localization of the Cj0371 protein were carried out to confirm whether *cj0371* plays a role in the flagella system. This report provides important information for understanding *C. jejuni* virulence and brings unique insight into the evolution and function of this remarkable bacteria.

## Materials and methods

### Bacterial strains and culture conditions

The bacterial strains and plasmids used in this study are listed in Table 1. *C. jejuni* 11168 was used to generate the *cj0371* deletion mutant and complement strain. *C. jejuni* were routinely grown on Campy blood-free selective medium (CCDA; Oxid Ltd., UK) or Mueller-Hinton agar (MH; BD Ltd., USA) microaerobically [85% N_2_(v/v), 10% CO_2_(v/v), and 5% O_2_(v/v)] in a jar at 42°C. *E. coli* DH5α that was used for cloning purposes was routinely cultured in Luria-Bertani (LB) medium at 37°C overnight. When necessary, culture media were supplemented with appropriate antibiotics, chloramphenicol (20 μg/ml), kanamycin (50 μg/ml), or *C. jejuni* supplement (SR0204E, Oxid Ltd., Basingtoke, UK).

**Table 1:** **Bacterial strains and plasmids used in this study**.

**Strains or plasmids**	**Genotype**	**Source or reference**
**STRAINS**		
***E.coli***		
DH5ɑ	*endA1 hsdR17* (*r*^−^_*K*_ m^+^_*K*_) *supE44 thi-1 recA1 gyrA relA1* Δ(*lacZYA-argF*)*U169 deoR* [Φ80*dlac* (*lacZ* Δ*M15*)]	Invitrogen
DH5ɑ-pRK2013	Conjugation helper strain, DH5ɑ containing plasmid PRK2013	Alkemade et al., 2003
***C. jejuni***		
*C. jejuni* 11168	*C. jejuni* NCTC11168	National Collection of Type Cultures
*C. jejuni* Δ *cj0371*	*C. jejuni* NCTC11168 *cj0371::cm*^r^	This study
*C. jejuni* Δ *cj0371*+	*C. jejuni* NCTC11168 *cj0371::cm*^r^ (pOUA18-P*metK*-*cj0371*)	This study
**PLASMIDS**		
pMD-19T	T clone vector, Aamp^r^	Takara
pMD-20T	T clone vector, Aamp^r^	Takara
pUOA18	*E. coli*-*Campylobacter* shuttle vector, *Cm*^r^	
pUOA18-P*metK*	*E. coli*-*Campylobacter* shuttle vector with *metK* gene promoter	This study
pRY107	Kan^r^ resistance plasmid, *E. coli*-*Campylobacter* shuttle vector	
pRK2013	*E. coli*-*Campylobacter* shuttle helper plasmid	Alkemade et al., 2003
pRY107-*egfp*	*E. coli*-*Campylobacter* shuttle vector with *egfp* gene	This study

### Bioinformatics analysis

ProtParam was used to analyze the physicochemical properties of the *Cj0371* protein (http://web.expasy.org/protparam/). *Cj0371* has one signaling peptide and one transmembrane structure predicted by CBS Prediction Servers (http://www.cbs.dtu.dk/services/). EMBL String was used to find homologs of *Cj0371* and investigate *cj0371* genetic structure. All BLAST searches were conducted within BioEdit and a BLAST database was also made using BioEdit (Reuter et al., 2015).

### Generation of *C. jejuni* 11168 Δ*cj0371* and *C. jejuni* 11168 Δ*cj0371*+

Deletion of the *cj0371* gene was achieved by homologous recombination using a suicide vector containing a homologous sequence on either side of the *cj0371* gene as described previously (Miller et al., 1988; Guo et al., 2008; Javed et al., 2012; Handley et al., 2015). Briefly, the *cj0371* open reading frame, including 200 bp of upstream sequence and 180 bp of downstream sequence, was amplified by PCR from *C. jejuni* 11168 using primers *cj0371*-F1 and *cj0371*-R1 (Table 2). Then, the fragments were cloned into pMD-19T to generate pMD-19T-*cj0371. Pst* I was the only restriction site in 338~393 bp of *cj0371* open reading frame. The *Cm*^*r*^ cassette was amplified from pUOA18 using *Cm*^*r*^-F and *Cm*^*r*^-R primers and ligated to pMD-19T to generate pMD-19T-*Cm*^*r*^. Using *Pst* I to digest pMD-19T-*cj0371*, pMD-19T-*Cm*^*r*^, and the *Cm*^*r*^ fragment was ligated into pMD-19T-*cj0371* to generate the suicide plasmid pMD-19T-*cj0371*-*Cm*^*r*^. Transferring the suicide plasmid into DH5α cells, transformants were selected on plates containing Ampicillin by blue-white colonies selection. After screening, one clone containing the cassette inserted in the same orientation as *cj0371* was used to mutate *C. jejuni* 11168 by electroporation and allelic exchange. One *Cm*^*r*^ mutant was selected on MH agar containing 20 μg/ml of chloramphenicol. The mutation was confirmed by PCR analysis and nucleotide sequencing.

**Table 2:** **Primers used in this study**.

**Primers**	**Primer sequences(5′–3′)**	**Amplicon size**	**Restriction sites**
*cj0371*-F1	GTGCAAGCTTCGCAAAAAACAAAAAATT	1.0 kp	*Hind* III
*cj0371*-R1	GCGCGAATTCACTTCTTGCGCTGCAGCA		*EcoR* I
*cm*^r^-F	GATCTGCAGTGGAGCGGACAACGAGTAAA	1.1 kp	*Pst* I
*cm*^r^-R	GATCTGCAGTCAGTGCGACAAACTGGGATT		*Pst* I
*cj0371*-F2	ATAGGATCCATGAAAAAAATCAAAAAA	606 bp	*BamH* I
*cj0371*-R2	GTAGAGCTCTTAAGAGCCAAAAGAAGA		*Sac* I
*cj0371*-F3	CGCGGATCCATGAAAAAAATCAAAAAA	603 bp	*BamH* I
*cj0371*-R3	ATCCGGATCCAGAGCCAAAAGAAGAAC		*BamH* I
P*metK*-F	GCGTCTAGATAATTTCCGCTTGAAAGAGCA	592 bp	*Xba* I
P*metK*-R	CGCGGATCCTCCTTTCATTTAAAATGAACC		*BamH* I

Complementation of the *cj0371* mutation in *C. jejuni* Δ*cj0371* was carried out as descried previously (Zeng et al., 2009; Chandrashekhar et al., 2015). The *cj0371* coding sequence along with its promoter region was amplified by PCR using specific primers (cj0371-F2 and cj0371-R2 which contain *BamH* I and *Sac* I site at 5′ ends, respectively) and cloned into pMD-20T to generate pMD-20T-*cj0371*. Using *BamH* I and *Sac* I to digest pMD-20T-*cj0371*, the fragments were cloned into pUOA18-P*metK*. The sequence of pUOA18-P*metK-cj0371* was confirmed by PCR. Then, following digestion by *Xba* I and *Sac* I, P*metK-cj0371* was cloned into pRY107, which was then digested by the same enzymes. P*metK* is the promoter region of *metK* gene. It was utilized as a strong promoter to ensure *cj0371* overexpression. The recombinant plasmids were transformed into *E. coli* DH5ɑ, and subjected to restriction analysis to confirm that they carried the desired sequence. The plasmids were introduced into *C. jejuni* Δ*cj0371* by biparental conjugation as described previously (Guerry et al., 1994). Transconjugants were selected on MH agar plates containing chloramphenicol and *Campylobacter* supplement (SR0204E Oxid Ltd., UK.). One transconjugant was selected and further confirmed by PCR to verify the presence of the wild-type copy of *cj0371* (Yao et al., 1993; Akiba et al., 2006).

### *In vitro* adhesion and invasion assay

The adhesion and invasion potentials of all strains were determined using *in vitro* adhesion and invasion assays as described earlier (Everest et al., 1992; Tareen et al., 2013). Caco-2 cells were seeded into 24-well plates at semiconfluency (~1 × 10^5^ per well) ~24 h prior to infection. When Caco-2 cells were grown to ~100% confluence, they were washed with PBS and inoculated with 1.0 × 10^7^ *C. jejuni*, a multiplicity of infection of 100. To investigate adhesion, the infected monolayer cells were incubated for 2 h to allow invasion to occur. Following the invasion period, wells for assaying adhesion were washed 3 times with PBS. Cells were lysed using 0.1% (v/v) Triton X-100 for 7 min at room temperature, serially diluted (10-fold) in PBS and 100 μl of each dilution was spread on a CCDA plate. The plates were incubated for 48 h at 42°C under microaerobic conditions after which colony forming-units (CFUs) were counted. To assess invasion, the *C. jejuni* suspension was removed after 2 h and the cells were washed 3 times with PBS before further incubation with culture medium supplemented with 100 μg/ml gentamicin. Subsequently, infected cells were rinsed with PBS and lysed with 0.1% Triton X-100; plating of the bacteria was performed as described above.

### Infant rabbit colonization assay

Litters of 1-day old New Zealand White infant rabbits were acquired from a commercial breeder (Jinlin infant rabbit farm, Nanjing, Jiangsu, China). The animal experimental design and protocols were approved by the Institutional Animal Care and Use Committee (IACUC) of Yangzhou University. The rabbit model was used as previously described (Ritchie et al., 2010, 2012). The infant rabbits were injected with cimetidine (50 mg/kg intraperitoneal injection) 3 h before orogastric inoculation with either 1 × 10^9^ cfu wild type *C. jejuni* 11168 or Δ*cj0371*, using a size 4 French catheter (Arrow International, Reading, PA). The *C. jejuni* to be tested were grown on MH agar plates for 24 h at 42°C and were harvested in sodium bicarbonate solution (2.5 g in 100 ml; PH 9), and the final concentration of inocula was adjusted by centrifugation (8 min,1000 rpm) to 5 × 10^9^ CFU/ml. After inoculation, rabbits were euthanized at fixed times after infection, 24 and 48 h. To count the number of *C. jejuni* CFUs in the cecum and colon, *C. jejuni* was plated on selective media. Cecum and colon samples were weighed and homogenized in 1 ml PBS, serially diluted, and spotted 20 μl on CCDA plates containing Cefoperazone (32 μg/ml) and Amphotericin (10 μg/ml) for enumeration of CFU per gram of tissue. The plates were incubated at 42°C miroaerobically. Some rabbits were not colonized by *C. jejuni* and these rabbits were excluded from all further analyses. The limit of detection was 50 CFU/ml or 50 CFU/g.

### Subcellular localization of Cj0371 protein

To determine where the Cj0371 carries out its function in *C. jejuni*, we used GFP tagged Cj0371 to investigate its subcellular localization by fluorescence microscopy. First, we constructed *C. jejuni* Δ*cj0371* expressing a functional green fluorescent protein of Cj0371. Using primers *cj0371*-F3 and *cj0371*-R3 (Table 2), we amplified *cj0371* without its termination codon from *C. jejuni* 11168, and cloned it into pMD-19T to generate pMD-19T-*cj0371*. *BamH* I was used to digest pMD-19T-*cj0371* and pUOA18-P*metK*, then the *cj0371* fragment was cloned into pUOA18-P*metK* to generate pUOA18-P*metK*-*cj0371*, following by cloning the P*metK-cj0371* fragment into pRY107-*egfp*. The recombinant plasmids pRY107-P*metK-cj0371*-*egfp* were then transformed into *E. coli* DH5ɑ and subjected to restriction analysis to confirm that they carried the desired sequence. The plasmids were introduced into *C. jejuni* Δ*cj0371* by biparental conjugation. Transconjugants were selected and confirmed as described above. The resulting strains were grown in MH liquid medium to OD_600_ of 0.4 and washed with PBS once. Ten microliters of the culture volume was loaded on slides, then spotted with 10 μl 4% paraformaldehyde in the bacterial suspension and observed on a Leica SP8 STED 3X confocal microscope.

### Motility plate assay and TEM imaging

The motility of mutant and complement strains was examined as previously described (Sommerlad and Hendrixson, 2007). Briefly, *C. jejuni* strains were grown on CCDA agar for 48 h in microaerobic conditions at 42°C. Then, the bacteria were resuspended in pre-warmed phosphate-buffered saline (PBS), and the optial density at 600 nm (OD_600_) of the bacterial solution to be tested was adjusted to 1.0. Each tested strain was spotted on the centers of duplicate MH plates and test tubes containing 0.4% agar. The plates were then incubated under microaerobic conditions at 42°C. After culturing for 48 h, the swarming diameter of the tested strains were compared to the wild-type *C. jejuni* 11168.

To examine the flagellar architecture of the mutants, 1%(wt/vol) ammonium molybdate-stained grids were used as previously described (Kalmokoff et al., 2006). *C. jejuni* strains were grown on CCDA agar plates, and diluted to an OD_600_ of 0.4. The bacteria were pelleted and resuspended in 2% glutaraldehyde (PH 7.0) for 1 h at room temperature, and samples were stained with 1% (wt/vol) ammonium molybdate-stained grids and visualized with a Tecnai 12 transmission electron microscope at an accelerating voltage of 120 kV.

### Assessment of growth

All *C. jejujni* strains were cultured on CCDA agar for 24 h in microaerobic conditions at 42°C. Bacteria was harvested from plates and transferred to MH broth. The OD_600_ of the bacterial cultures to be tested was adjusted to 1.0 and diluted 10-fold. The bacterial suspensions were then subpackaged into 8 anaerobic tubes with 4 ml in each tube and cultured at 37°C with shaking (180 rpm). Every 3 h, a tube of each strain was removed to observe the OD_600_.

### Chemotaxis assay

Using a modified hard-agar plug (HAP) procedure, we tested the chemotaxis of wild type and mutant *C. jejuni* (Hugdahl et al., 1988; Li et al., 2014). Each strain was grown on MH agar plates with 5% (v/v) sheep blood for 24 h at 42°C microaerobically. The bacteria were harvested with PBS, and the concentration was spectrophotometrically adjusted to 1 × 10^9^ CFU/ml. To prepare the HAPs, we made a 4% agar solution by dissolving 20 g of agar powder in 500 ml of PBS, autoclaving the solution, and added the test chemical in the 4% agar solution tempered at 60°C. Test chemicals were dissolved in PBS at 0.2 M and then filter sterilized (0.22-μm pore-size). The test chemical solution of 10 ml was added to 10 ml of the 4.0% agar solution, mixed, and poured into petri dishes (90 × 15 mm). After cooling, the agar was cut into 8 mm-diameter plugs (HAPs) with sterile, hollow, bullet casings (8-mm inner diameter). Five milliliters bacterial suspension was mixed with the same volume of heat-melted 0.8% agar in PBS at 42°C, and then poured into petri dishes (90 × 15 mm). HAPs containing a test chemical were placed with sterile toothpicks in soft agar. The plates were incubated microaerobically at 42°C for 6 h, and the diameters of accumulative bacterial rings toward each of the attractants in the plates were measured.

### Statistical analysis

Data analysis was performed using GraphPad. Statistical significance of the data was determined using Student′s *t*-test in cases, where only two data sets were compared. The values of *P* < 0.05 was considered statistically significant.

## Results

### Characterization of the *cj0371* mutant and complement

According to the results of PCR and sequencing, *cj0371* was disrupted by the insertion of the *Cm*^*r*^ sequence. The PCR and sequencing data also confirmed that the disruption of *cj0371* in *C. jejuni* Δ*cj0371* was complemented with the correct ORF compared to the wild type strain using the complementation vector. In this study, the sequences of the primers for the gene-disrupted or complemented mutant were derived from chromosomal DNA of *C. jejuni* 11168. Furthermore, we used an anti-Cj0371 monoclonal antibody (our group had made) to confirm that the Cj0371 protein in the mutant was undetectable. The complement mutant presented the Cj0371 protein at a higher level than the wild type strain (see the Supplementary Material).

### *cj0371* is a virulence-associated gene

**Figure 1:**
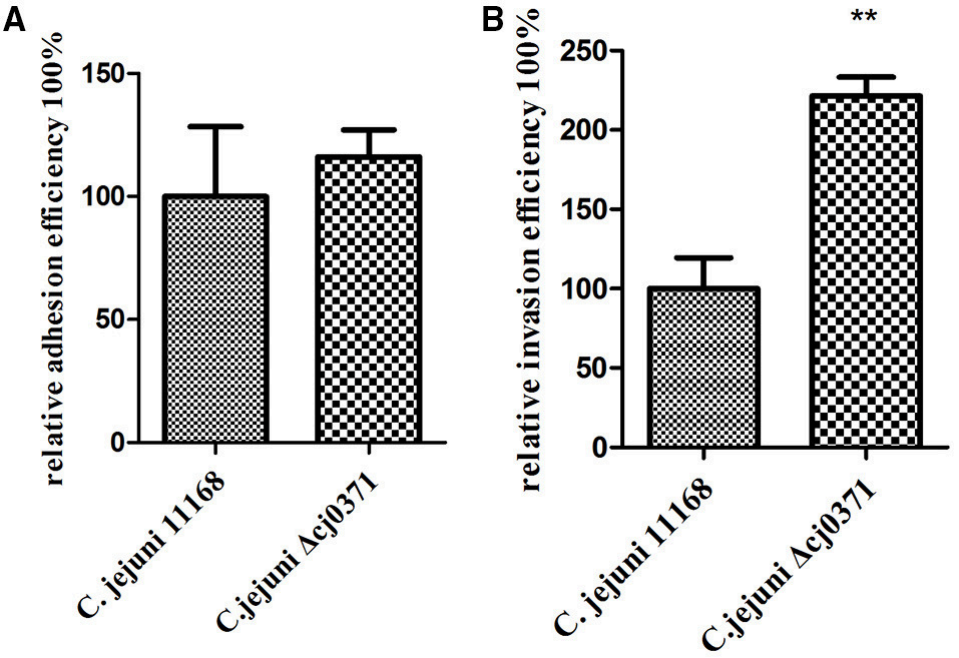
*****In vitro*** adherence and invasion assay of ***C. jejuni*** 11168 and ***C. jejuni*** Δ***cj0371*****. The adhesion phenotype of *C. jejuni* 11168 and *C. jejuni* Δ*cj0371* **(A)** and the invasion phenotype **(B)** were determined using Caco-2 cells. The adhesion ability of the *cj0371* mutant is similar to the wild type (*P* > 0.05), but compared to the wild type, *C. jejuni* Δ*cj0371* showed significantly increased invasion ability (*P* < 0.01). ^*^P ≤ 0.01; ^**^P ≤ 0.05; ^***^P ≤ 0.001.

**Figure 2:**
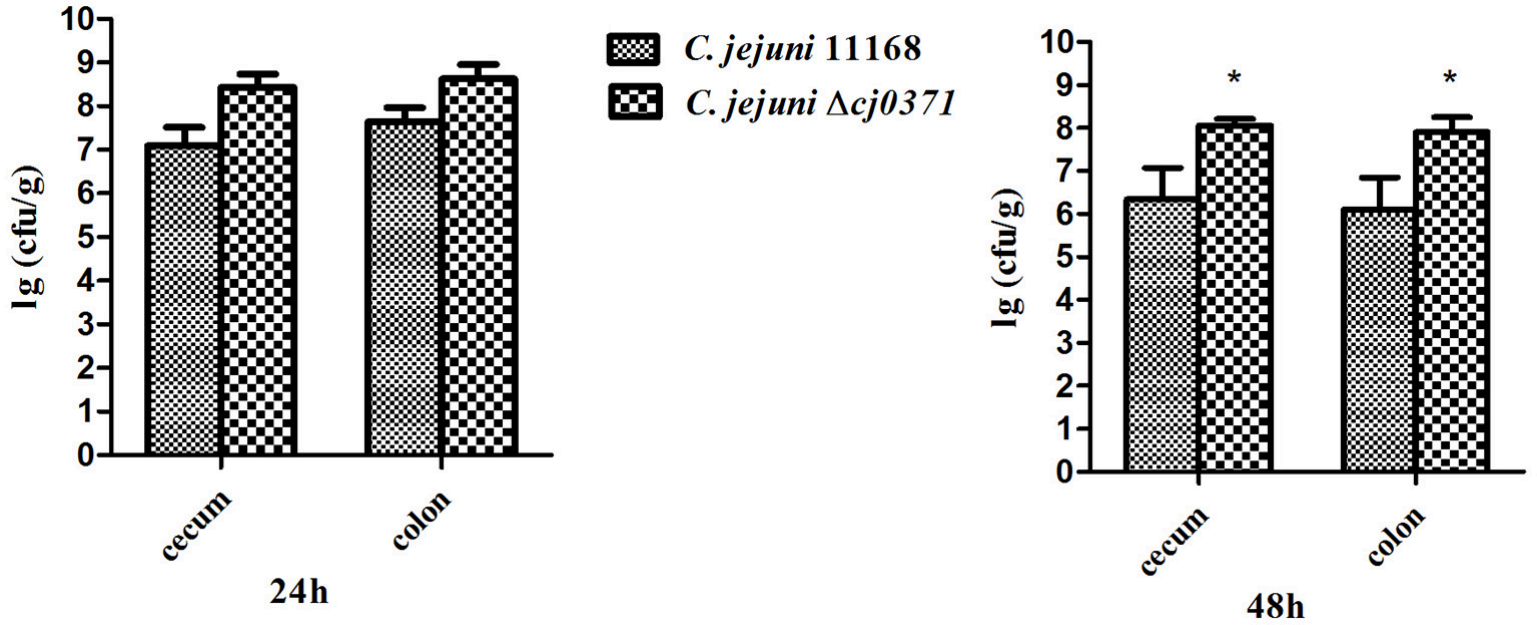
**Infant rabbit colonization assay of ***C. jejuni*** 11168 and ***C. jejuni*** Δ***cj0371*****. At 24 h PI, the colonization level of *C. jejuni* 11168 and *C. jejuni* Δ*cj0371* has no significant differences (*P* > 0.05). However, 48 h PI, the colonization level of *C. jejuni* Δ*cj0371* is significantly high than *C. jejuni* 11168 (*P* < 0.05). ^*^P ≤ 0.01; ^**^P ≤ 0.05; ^***^P ≤ 0.001.

The invasion ability of *C. jejuni* is an important pathogenicity-associated factor (Dasti et al., 2010). Many previous studies concluded that early mucosal damage is a result of invasion of *C. jejuni* into the epithelial cells (Field et al., 1986; Babakhani et al., 1993). First, we carried out an adhesion and invasion assay to examine the ability of *C. jejuni* Δ*cj0371* to infect Caco-2 cells compared to *C. jejuni* 11168. Importantly, we did not observe significant differences (*P* > 0.05) in adhesion ability between *C. jejuni* 11168 and *C. jejuni* Δ*cj0371* (Figure 1A), but *C. jejuni* Δ*cj0371* showed significantly increased invasion ability (*P* < 0.01) compared to *C. jejuni* 11168 (Figure 1B). Similarly, *C. jejuni* Δ*cj0371* exhibited slightly increased colonization ability (though without statistically significant differences) in infant rabbits 24 h after infection, and at 48 h post-infection, the colonization level of *C. jejuni* 11168 was significantly (*P* < 0.05) less than *C. jejuni* Δ*cj0371* (Figure 2). These results indicate that *cj0371* is a virulence-associated gene.

### *cj0371* localizes at *C. jejuni* cellular poles

**Figure 3:**
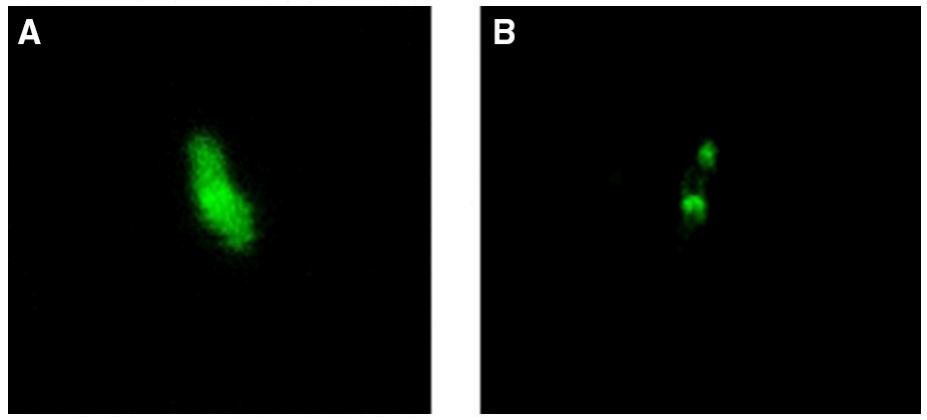
**Subcellular localization of the Cj0371 protein. (A)** *C. jejuni* Δ*cj0371* expressing GFP. **(B)** *C. jejuni* Δ*cj0371* expressing Cj0371-GFP proteins.

We constructed *C. jejuni* Δ*cj0371* expressing functional Cj0371-GFP and GFP, and observed its fluorescence using confocal microscopy. We found that Cj0371-GFP localized at the two *C. jejuni* poles (Figure 3). Given the polar localization of flagella in *C. jejuni*, we reasoned that this gene may play a direct or indirect role in the flagellar system. This observation led us to question a role for *cj0371* in flagellar biosynthesis and function.

### *cj0371* doesn't directly influence *C. jejuni* flagellar biosynthesis

**Figure 4:**
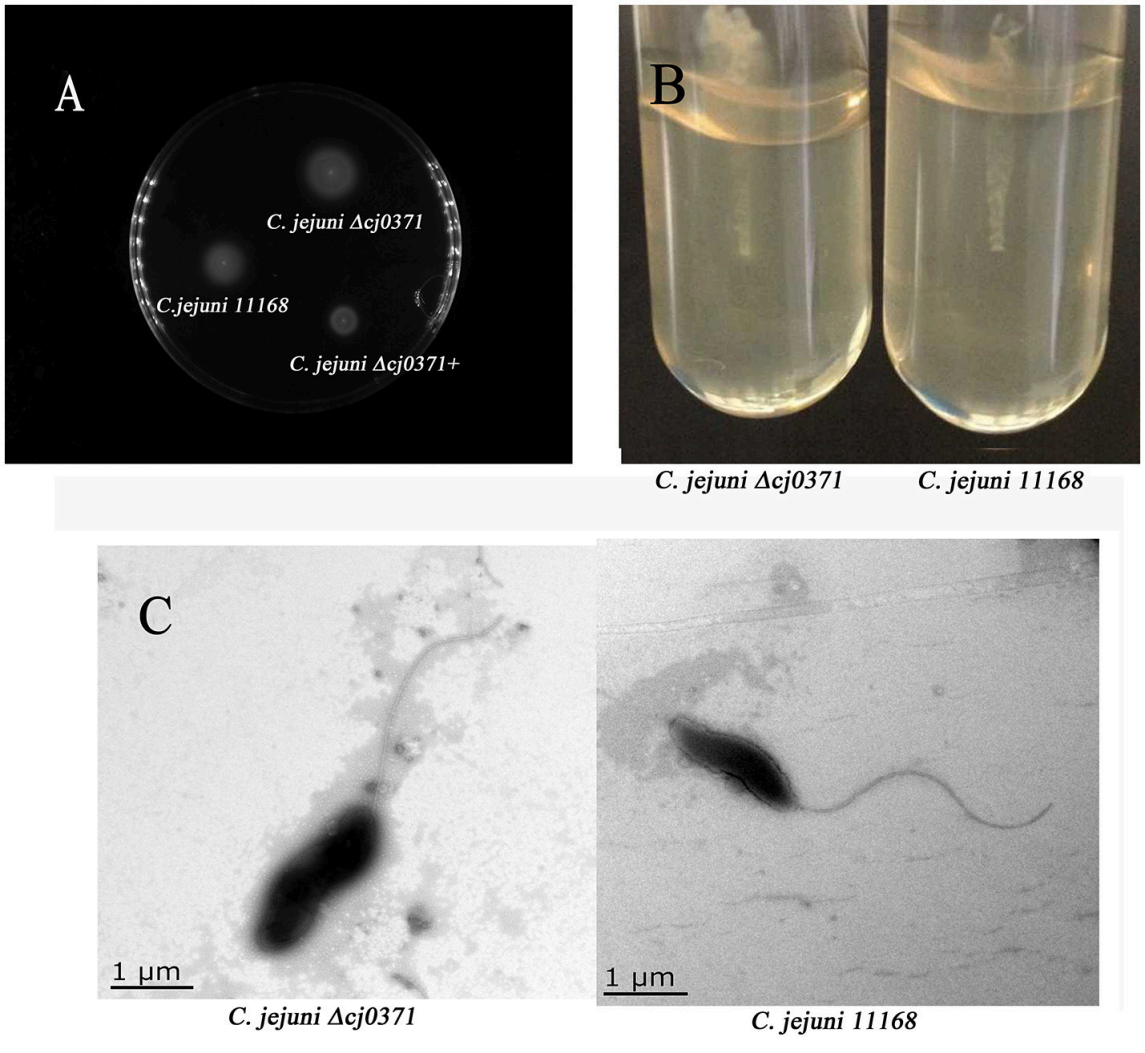
**Motility plate assay and TEM imaging. (A)** Motility analysis of *C. jejuni* 11168, the *cj0371* mutant and the complemented strain on soft agar. WT, wild type *C. jejuni* 11168-, *C. jejuni* Δ*cj0371*+, *C. jejuni* Δ*cj0371*+. **(B)** *C. jejuni* 11168 and *C. jejuni* Δ*cj0371* were cultured in a soft agar tube for 48 h to analyze motility. **(C)** Transmission electron microscopy analysis of *C. jejuni* 11168 and the *cj0371* mutant strain.

We used a soft-agar motility plate assay to compare the motility of *C. jejuni* Δ*cj0371, C. jejuni* 11168, and *C. jejuni* Δ*cj0371*+ (Figures 4A,B). Surprisingly, we found that *C. jejuni* Δ*cj0371* exhibited hypermotility and *C. jejuni* Δ*cj0371*+ showed a motility defect. Potentially, a motility defect phenotype could not be effectively shown by a soft-agar motility plate assay (Gao et al., 2014). So we observed the flagella of *C. jejuni* Δ*cj0371* and wild type *C. jejuni* by transmission electron microscopy (TEM; Figure 4C). However, we did not find any difference on the bacterial surface; *C. jejuni* Δ*cj0371* exhibited apparently normal flagella at its poles, indicating that *cj0371* is not directly associated with flagellar synthesis and assembly. We additionally quantified the proportion of bacteria exhibiting one or both poles with no polar flagella, but there was no difference between the mutant and wild type strain (data not shown).

### *cj0371* influences chemotactic behaviors of *C. jejuni*

**Figure 5:**
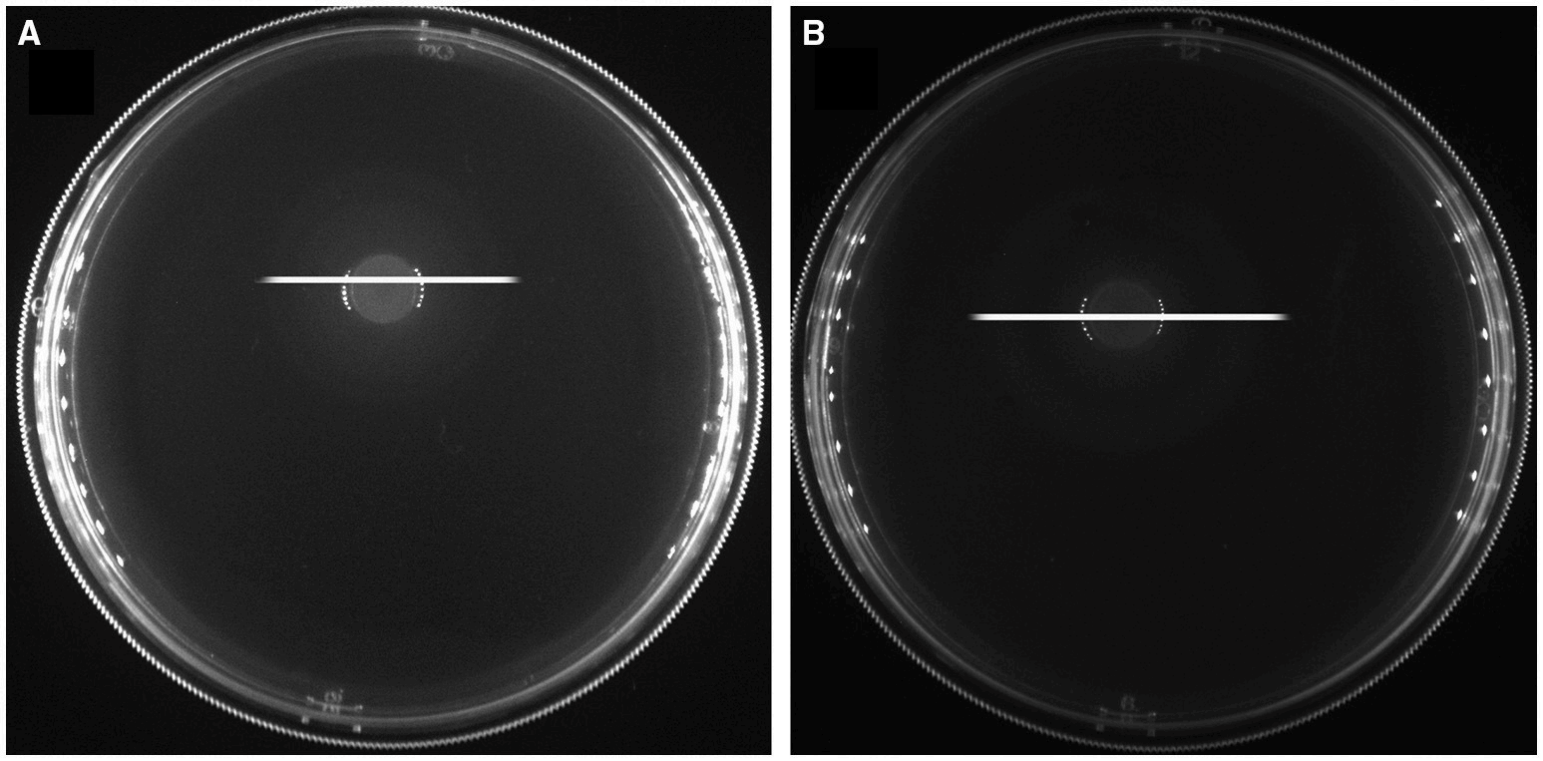
**Chemotaxis of the mutant and wild-type strain**. The zones of accumulation (chemoattractant) of *C. jejuni* Δ*cj0371* and *C. jejuni* 11168 after 6 h of incubation at 42°C. The plug contains 0.1 M DL-malic acid and the length of white lines are *C. jejuni* 11168 **(A)** and *C. jejuni* Δ*cj0371* **(B)** diameters of chemotaxis rings.

Previously, studies have speculated that *C. jejuni* use chemotaxis to reach particular milieu (Chang and Miller, 2006). We selected DL-malic acid, ketoglutaric acid and succinic acid, which are confirmed chemoattractants (Hugdahl et al., 1988), and utilized HAPs procedures to assay the chemotaxis of *C. jejuni*. We found the chemotaxis of *C. jejuni* Δ*cj0371* is stronger than *C. jejuni* 11168 (Figure 5 Table 3). Thus, we concluded that *cj0371* is associated with *C. jejuni* chemotaxis.

**Table 3:** **The diameters of chemotactic rings with different attractants**.

**Attractant (0.1 M)**	***C. jejuni* 11168 diameter of chemotactic ring (mm)**	***C. jejuniΔcj0371* diameter of chemotactic ring (mm)**
α-ketoglutarate	27	38
succinate	19	21
DL-malic acid	27	35

### Mutation of *cj0371* influences *C. jejuni* growth

**Figure 6:**
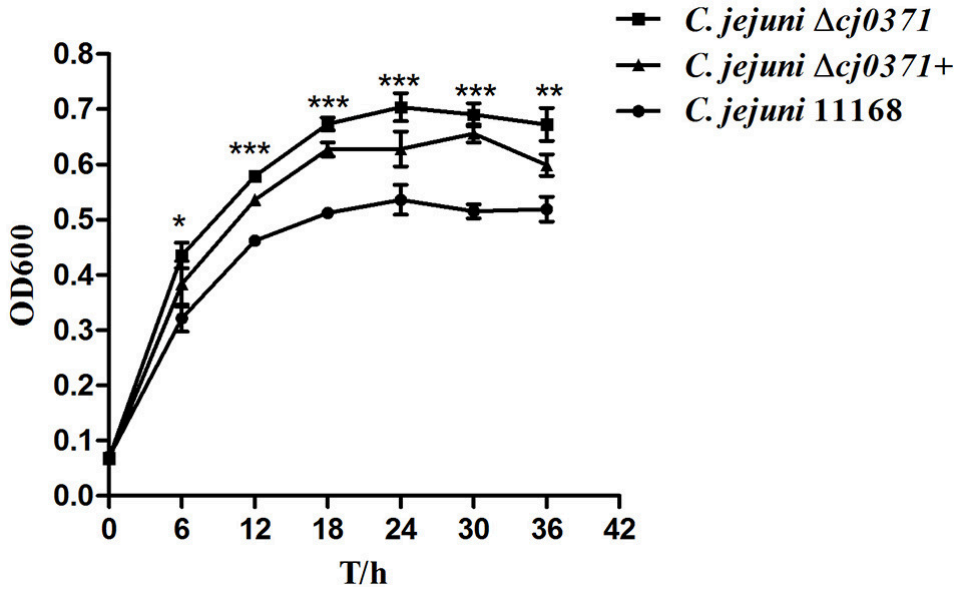
**Growth kinetics of ***C. jejuni*** 11168 and the ***cj0371*** mutant in MH broth**. The strains were cultured under microaerobic conditions at 42°C. At indicated time points, bacterial suspensions in anaerobic tubes were taken to measure OD_600_. The experiment was repeated three times, and the results of one representative experiment are shown. ^*^P ≤ 0.01; ^**^P ≤ 0.05; ^***^P ≤ 0.001.

To estimate *C. jejuni* Δ*cj0371* growth, we observed its growth curve compared to the wild-type *C. jejuni* 11168. We used anaerobic tubes to culture *C. jejuni*. According to the growth curves (Figure 6), *C. jejuni* Δ*cj0371* displays a growth increase compared with *C. jejuni* 11168 in MH liquid culture. *C. jejuni* Δ*cj0371* grows significantly faster than *C. jejuni* 11168 (6 h, *P* < 0.05; 12 h, *P* < 0.001; 18 h, *P* < 0.001; 24 h, *P* < 0.001; 30 h, *P* < 0.001; 36 h, *P* < 0.01). *C. jejuni* Δ*cj0371*+ grows slower than mutant, and *C. jejuni* 11168 has the slowest growth rate.

## Discussion

To our knowledge, *cj0371* was an unknown gene prior to this report. According to previous studies that investigated novel genes, we deleted the target gene from the wild-type and used a vector with a strong promoter to complement it. By observing the biological characteristics of the mutant and complement strain compared with the wild type, we expected to find a unique insight into the function of *cj0371*.

Adhesion and colonization of animal tissue by *C. jejuni* is an important step in establishing infection (Morooka et al., 1985). Mutation of *cj0371* was able to significantly increase invasion of *C. jejuni* into Caco-2 cells compared to wild-type *C. jejuni* (Figure 1). In addition, mutant strain colonized in distal gastrointestinal tract of infant rabbits better than wild type *C. jejuni*. These results suggested that *cj0371* is associated with *C. jejuni* virulence and that it is a negative control factor. To further prove our conclusion, we tested the expression level of *cj0371* when *C. jejuni* 11168 infected HD-11 cell, we found that *cj0371* showed up-regulated expression compared with the strain cultured *in vitro* (see the Supplementary Material). It is worth stressing that we selected infant rabbits as the experimental animals rather than poultry (such as chicken). One reason is that the infant rabbit colonization model has been successfully used for this pathogenic bacteria (publication pending), and another is that while poultry is the natural repository of *C. jejuni*, we could not successfully isolate *C. jejuni* from chickens after oral challenge. This could be explained by the presence of protective maternal antibodies in chick sera in the first week (Meunier et al., 2015).

Now, the recognized virulence factors of *C. jejuni* include motility, chemotaxis, colonization, adherence and invasion, cytolethal distending toxin (Cdt A,B,C; Dasti et al., 2010). *cj0371* was selected by IVIAT, so we speculated it was a virulence-associated gene. Its amino acid sequence is homologous to Cj8486_0361 (100%), which is defined as a putative flagellar motility protein [*C. jejuni subsp. jejuni* CG8486] (GenBank: EDK21732.1). Flagellar systerm and is energy burden associated with motility, so many mutations affecting unrelated physiological processes can indirectly affect motility (Nielubowicz et al., 2010; Neal-McKinney and Konkel, 2012). Because Cj0371 localizes at the *C. jejuni* cellular poles (Figure 3) and *C. jejuni* has a single flagellum at both poles, meanwhile Cj0371 has a signaling peptide and transmembrane domains, we speculated that *cj0371* may influence flagellar assembly and/or function. However, neither a soft-agar plate assay to observe the motility of the *cj0371* mutant and complemented strain, nor observation of the flagella on the surface of *C. jejuni* using TEM revealed significant evidence to confirm that *cj0371* is associated with flagellar synthesis and assembly.

*cj0371* does not influence flagellar assembly and biosynthesis, but it can increase the virulence of *C. jejuni*, so we speculated that it may control other factors such as chemotaxis, growth and metabolism. Another important finding of our work is that *cj0371* influences *C. jejuni* chemotaxis. Chemotaxis allows motile bacteria to travel toward a favorable niche or away from unfavorable conditions (Marchant et al., 2002). Cellular motility and chemotaxis have been implicated in the colonization and virulence of pathogenic bacteria and have a role in invasion and colonization of the host intestinal tract (Hartley-Tassell et al., 2010). To examine the chemotactic behaviors of *cheB, cheR*, and *cheBR* mutants, Kanungpean et al. carried out a semisolid agar motility assay (Kanungpean et al., 2011). In other worlds, motility is not only regulated by flagella but also controlled by chemotactic factors. The bacteria use a signaling cascade of protein phosphorylation and dephosphorylation reactions to control bacterial motors in response to environmental chemical changes (Miller et al., 2009). The direct association of the chemotaxis system with the flagellar apparatus affects the bacterial motility of *C. jejuni* (Zautner et al., 2012). In our study, the result of the motility assay is consistent with chemotaxis assay. Hypermotility and increased chemotaxis resulting in enhanced invasion and colonization seems reasonable. Including the data from the growth kinetic assay, hyperchemotaxis may allow *C. jejuni* Δ*cj0371* to travel toward a favorable niche, which benefits its growth. As for the *cj0371* complement growing faster than the wild-type *C. jejuni*, we propose that despite driving expression of the gene with a strong promotor, this may not result in overexpression in liquid medium. Of course, these conclusions are still inferences that need to be corroborated with further research.

Based on the results of invasion and colonization experiments, we discerned *cj0371* is a virulence-associated gene. However, the *cj0371* mutant has an increased capacity for invasion and colonization, so we conclude that it plays a negative role in pathogenicity, which is expected to be suppressed usually during infection. But *cj0371* was identified by IVIAT screening, suggesting this gene was induced during host infection. Actually, the genes screened by IVIAT are virulence-associated genes, including classical virulence genes and other virulence-associated genes (Rollins et al., 2005). We present data that collectively is not enough to prove *cj0371* is a typical virulence gene. So we temporarily call it virulence-association gene. This study presents an insight into the pathogenic mechanisms of *C. jejuni* and provides a keen interest to pursue in further study. In future studies, we intend to use co-immunoprecipitation to discover the protein that interact with Cj0371, followed by identification of interacting protein by liquid chromatography-mass spectrometry analysis. Through uncovering protein interactions we hope to further elucidate the function of Cj0371.

## Author contributions

XD as the first author, she participate to do all of the work including statistical analysis and this manuscript also was written by her. NW made the mutant strain and complement strain. FR and HT also participate to do part of the work. XJ and JH are tutor.

### Conflict of interest statement

The authors declare that the research was conducted in the absence of any commercial or financial relationships that could be construed as a potential conflict of interest.
