# Supplementary material for: cj0371: A Novel Virulence-Associated Gene of Campylobacter jejuni
Source: Front Microbiol. 2016 Jul 14;7:1094. doi: 10.3389/fmicb.2016.01094 (PMC4944492; doi:10.3389/fmicb.2016.01094)

The construction process of the suicide plasmid pMD19T-*cj0371*-*Cm^r^*


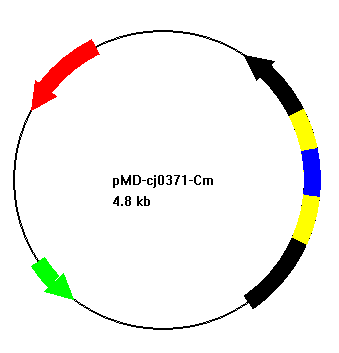


ori

*Ampr*

1

lacZ

MCS

*Past* I

*Past* I

*Hind* III

*EcoR* I I

*Past* I

*Past* I


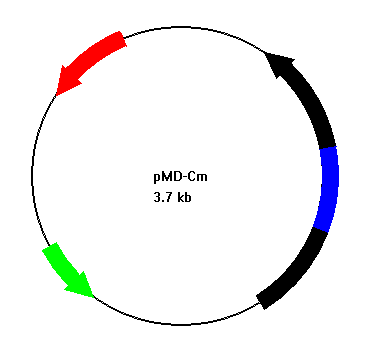


*Cmr*

*Ampr*

ori

1

lacZ

MCS


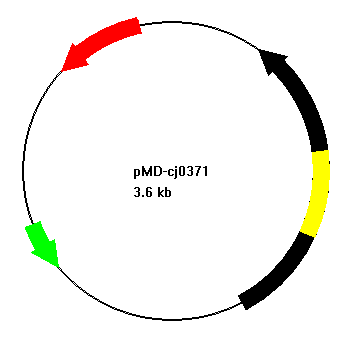


1

*Ampr*

ori

*Hind* III

*EcoR* I

*cj0371*

lacZ

MCS

The construction process of the complement plasmid pRY107-P*metK*-*cj0371*.

*Hind* III


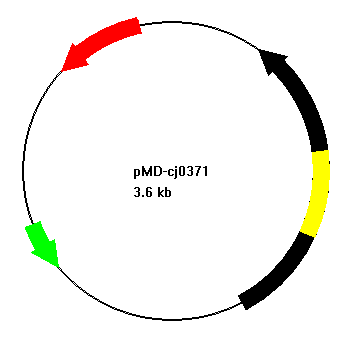


*cj0371*

lacZ

MCS

*Ampr*

ori

*EcoR* I

1


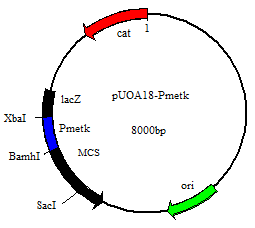


8.0kb


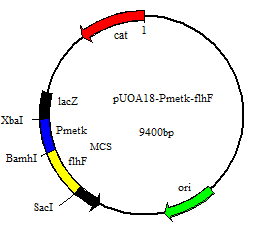


*cj0371*

8.6kb


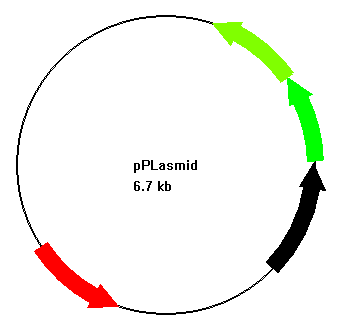


oriT

F1 ori

lacZ

MCS

Km

1

*Sac*I *Xba* I

pRY107


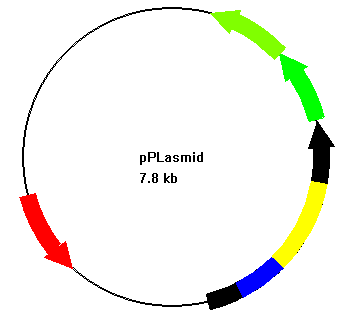


lacZ

MCS

ori*T*

F1 ori

Km

1

*Sac*I

*BamH* I

*Xba* I

The construction process of the complement plasmid pRY107-P*metK*-*cj0371-egfp*.

*BamH* I


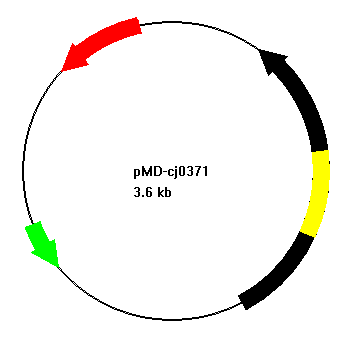


*cj0371*

lacZ

MCS

*Ampr*

ori

*BamH* I

1


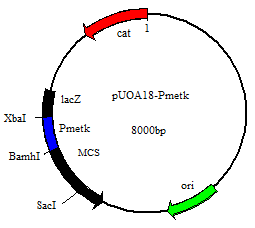


8.0kb


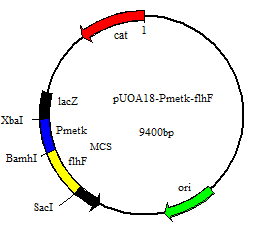


*cj0371*

8.6kb

*BamH* I

*BamH* I


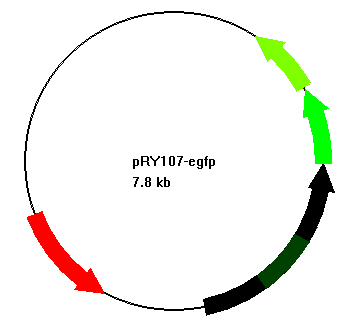


1

oriT

F1 ori

lacZ

MCS

*egfp*

Km

*BamH* I


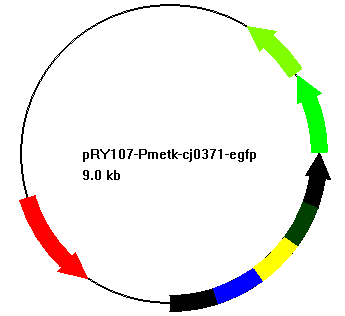


oriT

F1 ori

lacZ

egfp

MCS

Km

1

*BamH* I

*BamH* I

*Xba* I


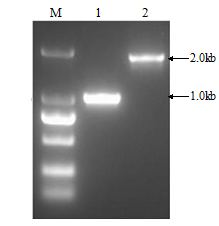


FIG 1 PCR identification of *cj0371* mutant strain

Lane M：DL2000 DNA marker

Lane 1：PCR identification of NCTC11168 witht *cj0371*-F1/R1

Lane 2：PCR identification of *cj0371* mutant strain with *cj0371*-F1/R1


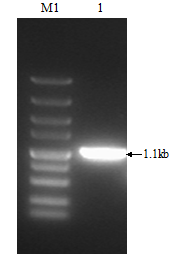


FIG 2 PCR identification of *cj0371* complementary strain

Lane M1：DL5000 DNA marker

Lane 1：PCR identification of *cj0371* complementary strain with P*metK*-F/*cj0371*-R2

FIG 3 Detection expression of Cj0371 protein by Western blot

Lane 1：NCTC11168 whole-cell protein

Lane 2：*cj0371* complementary strain whole protein

Lane 3：*cj0371* mutant strain whole protein


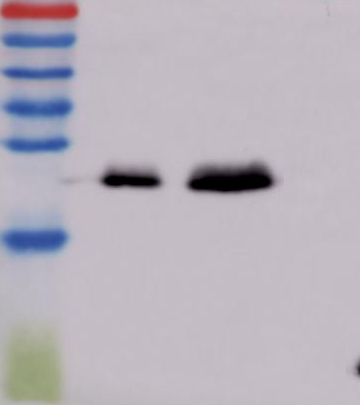


M 1 2 3

70KD

55KD

40KD

35KD

25KD

15KD

10KD


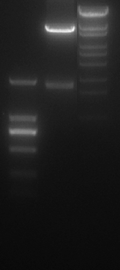


6750bp

1.800bp

FIG 4：Analysis the plasmid pRY107-P*metK*-*cj0371*-*egfp* with restriction enzymes.

Lane M1: DL2000 DNA marker

Lane M2: λ-EcoT 14Ⅰ digest marker

Lane 1: pRY107-(P*metK*+*cj0371*+*egfp* )/

pRY107 6750bp/P*metK*-*cj0371*-*egfp* 1800bp

M1 1 M2

FIG 5 When *C. jejuni* 11168 infected HD-11 cell, *cj0371* showed up-regulated expression compared with the strain cultured *in vitro.*


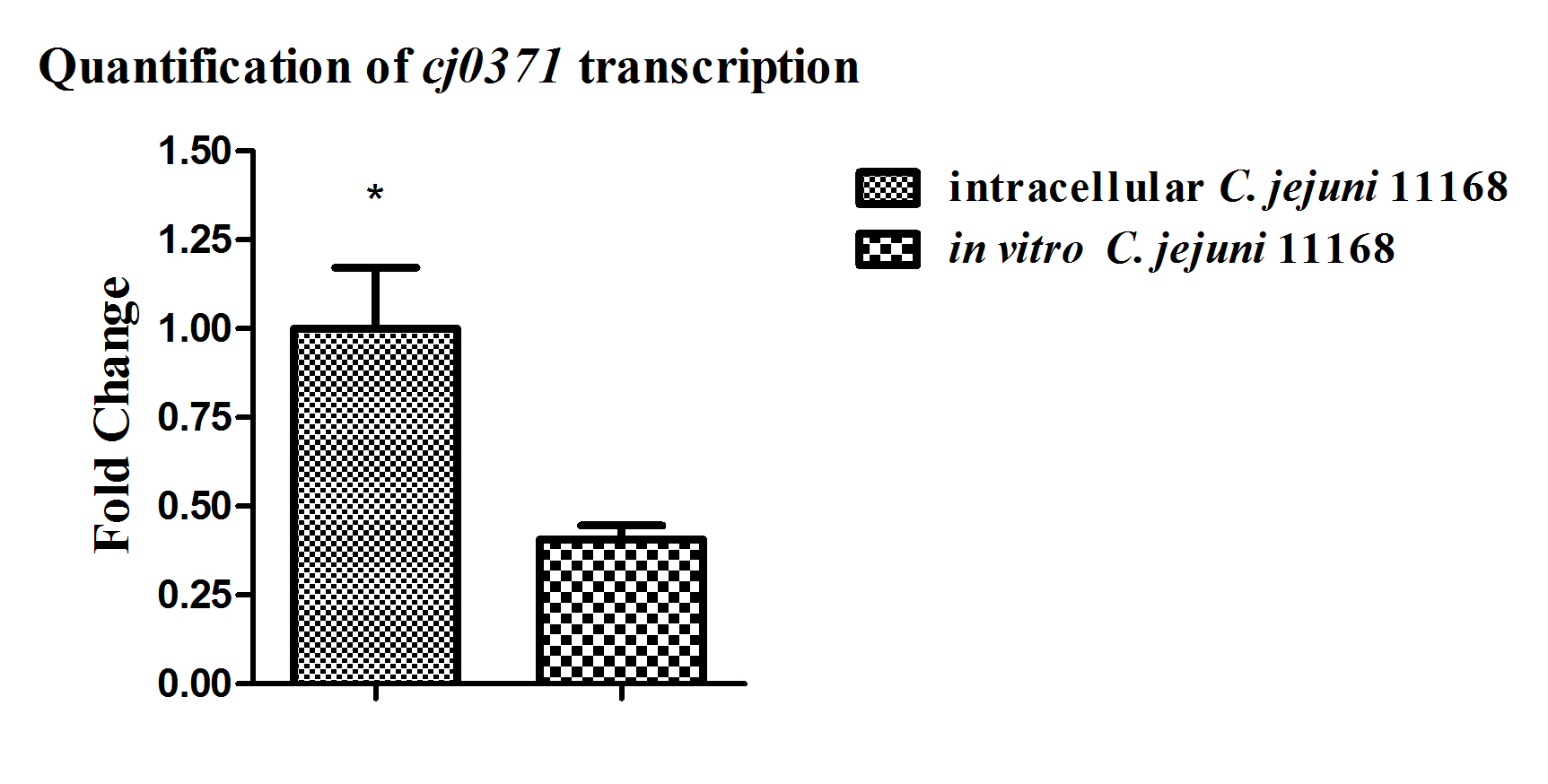

Supplement: Supplementary file 1 [file DataSheet1.DOC]
